# Lessons learned from the Philadelphia Collaborative Preterm Prevention Project: the prevalence of risk factors and program participation rates among women in the intervention group

**DOI:** 10.1186/s12884-014-0368-0

**Published:** 2014-11-01

**Authors:** David A Webb, Leny Mathew, Jennifer F Culhane

**Affiliations:** Children’s Hospital of Philadelphia, National Children’s Study Center, 3535 Market Street, Philadelphia, PA 19104 USA; Department of Pediatrics, University of Pennsylvania School of Medicine, and Department of Adolescent Medicine, Children’s Hospital of Philadelphia, 3535 Market Street, Philadelphia, PA 19104 USA

**Keywords:** Prematurity, Preterm birth, Pregnancy, Perinatal periods of risk, Health care participation, Infant mortality, Preventive care, Access to care, Utilization of care, Preconception care

## Abstract

**Background:**

Women who deliver preterm infants are at a much greater risk for repeating a preterm birth (PTB), compared to women without a history of PTB. However, little is known about the prevalence of the risk factors which account for this markedly increased risk. Moreover, little or nothing is known about the feasibility of providing treatments and services to these women, outside of the context of prenatal care, during the inter-conception period, which provides the best opportunity for successful risk-reduction interventions.

**Methods:**

The Philadelphia Collaborative Preterm Prevention Project (PCPPP), a large randomized control trial designed to identify and reduce six major risk factors for a repeat preterm birth among women immediately following the delivering of a preterm infant. For the women assigned to the PCPPP treatment group, we calculated the prevalence of the six risk factors in question, the percentages of women who agreed to receive high quality risk-appropriate treatments or services, and the of rates of participation among those who were offered and eligible for these treatments or services.

**Results:**

Urogenital tract infections were identified in 57% of the women, while 59% were found to have periodontal disease. More than 39% were active smokers, and 17% were assessed with clinical depression. Low literacy, and housing instability were identified in, 22 and 83% of the study sample, respectively. Among women eligible for intervention, the percentages who accepted and at least minimally participated in treatment ranged from a low of 28% for smoking, to a high of 85% for urogenital tract infection. Most PCPPP enrollees (57%) had three or more major risk factors. Participation rates associated with the PCPPP treatments or services varied markedly, and were quite low in some cases, despite considerable efforts to reduce the barriers to receiving care.

**Conclusion:**

The efficacy of individual level risk-reduction efforts designed to prevent preterm/repeat preterm in the pre- or inter-conception period may be limited if participation rates associated with interventions to reduce major risk factors for PTB are low. Achieving adequate participation may require identifying, better understanding, and eliminating barriers to access, beyond those associated with cost, transportation, childcare, and service location or hours of operation.

**Trial registration:**

ClinicalTrials.gov (NCT01117922)

## Background

Approximately 65-75% of infant mortality in the U.S. is related to preterm birth (PTB), and premature births account for almost half of all chronic and severe morbidity in young children [1,2]. Every year more than 450,000 babies in the U.S. are born prematurely (defined as less than 37 completed weeks of gestation), accounting for 11.6 percent of all live birth deliveries. Consequently, PTB has been the subject of a considerable amount of epidemiological and medical research. Unfortunately, the causes are still not well-understood, and thus the path to reducing PTB remains somewhat elusive [2].

The strongest predictor of PTB is a previous PTB. Women with a previous PTB are between 2 to 5-fold greater risk for a second PTB, compared to women with a previous full term delivery [3]. Moreover, the earlier the gestational age of a PTB, the greater the risk for a repeat PTB. As many as 50% of women with a prior history of PTB occurring at or before 32 weeks gestational age (GA) have a subsequent PTB [3,4].

Past efforts to reduce PTB have been largely unsuccessful, perhaps because such efforts target a single risk factor, and the corresponding services or interventions have been offered only during the course of pregnancy. What may be needed, therefore, are more comprehensive, long term interventions, beginning prior to conception and thus outside the context of traditional prenatal care [2,5,6].

In light of the extremely high rate of repeat preterm delivery, the most obvious target group for inter-conceptional risk reduction is women with a history of preterm birth. Unfortunately, little is known about this manifestly high risk group in terms of the degree of willingness or ability to participate in appropriate interventions or services in the postpartum or inter-conceptional period.

Presented here are the result of an analysis of data from the Philadelphia Collaborative Preterm Prevention Project (PCPPP), a large randomized control trial designed to identify and reduce risk factors for repeat preterm births among women who previously delivered moderately to severely premature (<35 weeks gestation) infants. PCPPP data were examined to determine both the prevalence of the major risk factors for PTB among enrollees, the frequency with which enrollees agreed to treatment, as well as the actual participation rates pertaining to each of the intervention arms of the study.

### The Philadelphia collaborative preterm prevention project

The results from both epidemiological and biological studies suggest that PTB may be a function of a more generalized pro-inflammatory process that increases the overall likelihood that some women will deliver prematurely [2,7-9]. Infections such as periodontal disease [10], urogenital tract infections [7], bacterial vaginosis and other sexually transmitted diseases [11], and pneumonia and influenza [2] are in fact associated with the increased risk of PTB. For that reason the core PCPPP intervention protocol focused on the social, behavioral and medical factors that may contribute to an increased risk of PTB through an inflammatory pathway or systemic inflammation (SI) -- specifically, smoking, depression, infectious disease burden, periodontal disease and maternal stress. A more detailed discussion of the justification for organizing the intervention protocol of PCPPP around SI-related factors has been published elsewhere [12,13]. An explanation of, and data pertaining to, the randomization process and the overall recruitment and retention of the women who enrolled in the PCPPP trial are also available in that publication. Systematic and continuous data collection and risk assessment were an integral part of the PCPPP study design with the objective of continuously identifying, referring and providing treatment for all women with any of the risk factors associated with SI noted above.

As described in more detail below, presence of the risk factors was determined using widely accepted medical procedures, or by way of standard social/behavioral assessments, administered by appropriately trained, professional or licensed staff. In addition, a comprehensive set of strategies were developed and implemented in order to eliminate barriers to receiving treatment and services, offered as a complement to intervention arms of the PCPPP trial. These strategies were in fact intended to maximize participation and included, but were not necessarily limited to: providing treatment or services completely cost-free; reimbursement for any costs related to transportation to and from clinical sites, including use of tokens and cab vouchers, or door-to-door, pick-up and drop-off car service provided by staff; free childcare; flexible clinical hours, including evening and weekend hours; and in home-visits when appropriate. In addition, PCPPP staff routinely followed-up with women regarding missed appointments. Underlying these measures was recognition of high thresholds of participation as a prerequisite for effective risk reduction, and for our ability to evaluate the effectiveness of the PCPPP interventions designed to reduce repeat preterm birth rates

## Methods

### Data

All women who were identified to us by the hospital, or by the Pennsylvania Department of Health using their electronic birth record reporting system, as having delivered at 35 weeks gestation or less were approached by PCPPP staff for permission and written consent to enroll in the study. From September of 2004 through August of 2008 the PCPPP team recruited from twelve Philadelphia hospitals providing 70% of the obstetric services in the region. Eligibility for enrollment was defined as: 1) delivery of singleton infant at <35 weeks of gestation; 2) English or Spanish speaking; 3) Philadelphia area residency; and 4) *not* receiving operative sterilization before discharge from the hospital. A total of 1,450 women met the eligibility criteria for PCPPP and were approached for enrollment. Of those 324 refused and 1,126 women agreed to enroll in the PCPPP trial. There were no significant sociodemographic differences between those who agreed and those who refused to enroll [12]. Of those who agreed to enroll 565 were randomized into the intervention group and 561 into the control group. All women who enrolled were asked to attend study visits and repeatedly assessed at 1, 6, 12, 18 and 24 months postpartum. Women randomized into the treatment group were regularly assessed for the presence of the pre-specified risk factors and invited to avail themselves of the state of the art treatment and services offered as part of the PCPPP protocol, which are described in detail below. *Consequently, in all cases, and for the purposes of the analyses described below, the “prevalence” of a risk factor refers to period prevalence. In other words, if at any time during any study visit an enrollee was assessed as having a particular risk factor, that enrollee was classified and counted as being at-risk for the risk factor in question. Similarly, treatments and services were offered based on need; specifically, interventions were offered based on assessments made over the entire course of the study visit schedule. Thus enrollees were continuously assessed and offered risk-appropriate treatments and services—initially when the risk factor was first identified, if the risk factor persisted based on any subsequent study visit, or if the risk factor re-appeared during any subsequent study visit.* Women who were randomized into the control group were administered identical assessments as the intervention group, were informed of the results, and were referred to appropriate medical or social service providers in the community, if available, but received none of the on-site, enhanced services or treatments funded by the PCPPP. Ethical approval to conduct the study was granted by the Institutional Review Board of Drexel University, Philadelphia Department of public Health, and the University of Pennsylvania.

A total of 471 women in the intervention group received at least one complete follow-up clinical assessment, and it is those women who comprise the analytical sample for the findings presented here. Based on a preliminary analyses of survey data collected at the time of enrollment we found that women assigned to the intervention group but who were lost to follow up after enrollment (n = 94) were not significantly or substantively different from the 471 women in the study sample, in terms of race/ethnicity, income, age, education, insurance status, birth weight or gestational age of the infant (Not shown; data provided upon request).

### Study variables

Key variables used in the analysis included 1) the presence or prevalence of risk factors for PTB/repeat PTB discussed above, which determined participant eligibility for treatment; 2) rates of acceptance by participants for risk-appropriate treatment; and 3) minimal participation rates in the treatment protocols.

1. *Determination of the presence/ prevalence of risk factors.*
  A summary of the protocol used for determining the presence of each risk factor and the corresponding intervention is presented in Table 1. Briefly stated:
  Urogenital tract infections. Biological samples were obtained by professional staff; presence of infection was based on result of standard laboratory testing. Medically appropriate antibiotic treatment was offered to all women who tested positive for infection.
  Periodontal disease. Periodontal assessment was completed by a licensed dental hygienist using the American Dental Association’s Periodontal Screening and Recording (PSR) questionnaire [14]. Women screening positive were sent to DDM/periodontist for clinical confirmation of periodontal disease and the development of a treatment plan which included scaling, root planing and/or extraction when appropriate.
  Smoking. Standard questionnaires administered by trained staff; interventions included range of acceptable treatments, including supportive counseling, nicotine patch therapy or Wellbutrin, or a combination of the above.
  Depression. Administration of the Center for Epidemiologic Studies Depression Scale (CES-D), a commonly used screening instrument for depressive symptomatology with known reliability and validity [15,16]. Women with CES-D scores of 16 or greater were considered to have depressive symptomatology and asked to complete the The *Structured Clinical Interview for DSM-IV* Dissociative Disorders (SCID), widely used in research settings in order to diagnose depression [17]. The SCID was administered by appropriately-trained and licensed professional study personnel. Those determined to have major depressive disorder were offered cognitive behavioral therapy (provided by a Masters prepared, licensed social worker or licensed psychologist), or psychopharmacology (provided by a staff psychiatrist) -- or both.
  Low literacy. A Standardized test, the Short Test of Functional Health Literacy in Adults (S-TOFHLA) or the Test of Adult Basic Education (TABE), commonly used instruments for assessing health literacy [18]. Both the S-TOFHLA and the TABE were administered by licensed social workers. The interventions for those determined to have low health literacy were individually-tailored and provided by professional adult educators.
  Housing instability. Completion of a comprehensive assessment of living arrangements, affordability, safety and threat or likelihood of eviction based on face to face interviews. The interventions for housing instability were individually-tailored, contingent on need, and provided by a licensed social worker experienced with resolving housing crises commonly occurring in lower income populations.
2. *Acceptance rates.* Participants in the intervention group were classified as having accepted treatment if, after being informed of their conditions, they consented in writing to the respective treatment options described above.
3. *Minimal participation rates.* A summary of how minimal participation was defined for this analysis is provided in last column of Table 1. In the majority of cases minimal participation was defined as at least one appropriate ‘follow-through’ by those participants who were identified with a risk factor, and therefore eligible for services or treatment. For periodontal disease and depression participation was defined as having made at least clinic visit for treatment. For smoking participation was defined as having attended at least one group or individual counseling session. For low literacy a women was considered to have participated if she attended at least one session designed to improve reading skills. In the case of urogenital infections an enrollee was classified as participating if she accepted the appropriate medication, which was provided on site and confirmed as taken appropriately through direct observation. For housing instability enrollees were classified as having participated if they accepted and received any tangible assistance to improve or stabilize their physical housing condition, including money to pay overdue rent or utility bills, relocation services, or mediation to resolve a landlord/tenant dispute.
  In brief, the consistent theme underlying the definition of “participation” was any confirmed use of treatment or risk-reduction services by eligible women; participation was, therefore, always defined minimally. The terms ‘participation’ and ‘minimum participation’ are used interchangeably here. The strategies used to address potential barriers to access to treatments or services are also presented in Table 1.

**Table 1:** **Definition of Risk factor identification, interventions, and participation**

	***Risk factor/Intervention***	***Definition of (Minimal) Participation/Strategies to address potential barriers to participation***
***Infection***	**Identification:** Positive, based on standard medical diagnostics for STD’s and urogenital tract infections, including Bacterial Vaginosis (BV), Chlamydia, Trichomoniasis, Gonorrhea, yeast infection, excessive bacteruria, and Syphilis. Tests were conducted at the centralized clinical setting; all lab results were reviewed by the study-trained laboratory coordinator.	**Participation:** Prescribed and provided (on site) standard medical treatment for all infections. with confirmation by direct observation that medication was taken
	**Treatment:** Follow-up and standard medical treatment for all conditions identified; supervised by the study medical team and free of charge.	**Strategies.** No cost treatment, flexible clinical hours, transportation to and from appointments, free childcare, follow up by social workers
***Periodontal disease***	**Identification.** Screened positive for clinical periodontal disease. Assessment, including soft tissue exam for oral cancer, Plaque Scores, Gingivitis Index Scores, Probing Pocket Depth, Bleeding Upon Probing, Clinical Attachment Level, and cementoenamel junction. Initial screening, was completed by the study registered dental hygienist and the presence of periodontal diseases was confirmed by a licensed periodontist.	**Participation** Attended at least one dental clinic appointment for scaling, root planning or surgery
	**Treatment:** Individually-tailored intervention including oral hygiene education and comprehensive clinical treatment for all conditions identified. Presence of periodontal disease was confirmed through x-ray and clinical exam by a DDM. Treatment for periodontal disease was provided by or under direct supervision of a periodontist/DDM, free of charge	**Strategies:** No cost treatment, flexible clinical hours, transportation to and from appointments, free childcare. appointment reminders. PCPPP staff was also available to accompany women to dental visits when requested.
***Smoking***	**Identification.** Reported smoking during pregnancy or postpartum period based on standardized questionnaire administered by study staff.	**Participation:** Attended at least one individualized or group counseling session, and/or received nicotine replacement therapy
	**Treatment:** Referral and follow-up for smokers, who were offered individually-tailored one-on-one cessation counseling and pharmacotherapy, including standard nicotine replacement therapy, and bupropion. One-on-one counseling was provided by certified smoking cessation counselor; pharmacotherapy was provided and prescribed by physician	**Strategies:** Free treatment, flexible hours, in home visits, free transportation to and from any scheduled appointment, free childcare., appointment reminders
***Major depressive disorder***	**Identification.** Screening with Center for Epidemiological Studies of Depression Scale (CES-D ≥16) followed by a diagnostic interview with the Structured Clinical Interview for DSM Disorders (SCID) for those with a positive screen. The SCID was administered by appropriately trained study physician or social worker.	**Participation:** Attended at least one therapy session and/or received medication for depression
	**Treatment:** Participants who were diagnosed with current major depressive disorder were offered medical treatments comprised of cognitive behavioral therapy, antidepressant psychopharmacology, following standardized protocols, or the combination of the two treatments. Women who declined these therapies were offered supportive counseling and problem solving training delivered by clinical social workers in home visits.	**Strategies:** Free treatment, flexible hours, free transportation to and from any scheduled appointment, free childcare, appointment reminders, in home visits.
***Low literacy***	**Identification.** Based on Short Test of Functional Health Literacy in Adults (inadequate and marginal: English and Spanish), Test of Adult Basic Education - Reading Locator (levels E&M; TABE) administered by study staff.	**Participation:** Attended at least one, one-on-one or classroom session to improve literacy
	**Treatment:** An individually tailored learner-driven intervention model was utilized using a contextual adult educational curriculum focused on building skills for navigating hurdles to maternal-infant care and family management/economics. Adult literacy skills were developed through working individually with professional adult educators on specific challenges faced by the participants and selected by them.	**Strategies** Free treatment, flexible hours, free transportation to and from any scheduled appointment, free childcare, appointment reminders
***Housing***	**Identification.** Based on comprehensive assessment of housing status and stability conducted by study staff. Those with problems indicating imminent eviction, in unsafe or unhealthy living environments were considered as	**Participation:** Received any form of financial, or relocation assistance in order to improve/stabilize housing situation, or mediation services to resolve landlord/tenant dispute
***Instability***	**Treatment:** Housing assistance, when appropriate, was provided in the form of cash grants for down payments or back rent, relocation services, or resolution of landlord/renter disputes, provided under the direction of a MSW, with experience in resolving housing-related issues	**Strategies.** Flexible hours, free transportation to and from any scheduled appointment, free childcare, appointment reminders

### Statistical analyses

The sociodemographic characteristics, including race/ethnicity, education, income, as well as nativity, marital and insurance status were collected at the time of by means of face-to-face surveys administered at the time of enrollment. Simple frequency distribution procedures were used to describe the study sample, and calculate eligibility, acceptance and participation rates.

Previous research has shown that minority and/or low socioeconomic status are associated with skepticism about, and unwillingness to participate in, medical research, including randomized control trials [19]. These same factors are also known to impede a woman’s ability to avail herself of health care and related services [20]. In order to determine the extent to which these factors were influencing participation rates for women in the study sample, we explored differences in risk factor prevalence, acceptance rates, and participation rates according to both race/ethnicity and insurance status (private vs. Medicaid or uninsured). A preliminary analysis of the data revealed that the pattern of relationships between prevalence, acceptance and participation rates on the one hand, and education and household income on the other, was very similar to that for insurance status. Consequently, for the sake of brevity and for purposes of illustration, only data pertaining to insurance status are presented here. Cross-tabulations were used to examine these differences, and chi-square values were calculated and used to identify any statistically significant relationships. (The Fisher’s Exact Test was substituted for chi-square if the expected value in any cell was less than 5). In cases where both insurance status and race/ethnicity were significantly correlated with the dependent variables (prevalence, acceptance, and participation) logistic regression analysis was conducted to determine if the associations were independently related to the dependent variables. The analysis was conducted using standard statistical programming software available in STATA 12.1 [21].

## Results

The sociodemographic and other characteristics of the PCPPP intervention group are shown in Table 2. Of the 471 women who were randomized into the intervention group and had at least one post-delivery clinical assessment, 93 percent were US born. In terms of race/ethnic composition, 72 percent were non-Hispanic Black, 10 percent were Non-Hispanic White, while the remaining 18 percent identified themselves as Hispanic or of some other race/ethnic group. More than 30% had not finished high school or completed a GED at the time of the index delivery. More than one-half (57.5%) reported household incomes of less than $30,000 per year, while about 70 percent were on Medicaid or were uninsured at the time of enrollment. The average age of mother at time of recruitment (delivery of a preterm infant) was 25.6 years, while the average gestational age of the index preterm birth was 30.2 weeks.

**Table 2:** **Description of the study sample**

**PCPPP Intervention Group: with at least one post-delivery clinical assessment**	
**All Women**	**N = 471 (100.0%)**
**US born**	440 (92.6)
**Race/Ethnicity**	
NH Black	335 (70.5)
NH-White	50 (10.5)
Hispanic/other	90 (19.0)
**Education**	
<HS	151 (30.8)
HS/GED	191 (40.2)
College or more	133 (28.0)
**Married**	84 (17.7)
**Income** < =10,000	101 (21.3)
10-30,000	178 (37.5)
>30,000	109 (22.7)
DK/refused	88 (18.5)
**Insurance status**	
Private insurance	143 (30.3)
Medicaid	317 (66.9)
Uninsured	14 (3.0)
**Age mean (sd)**	25.6 (6.6)
**GA mean (sd)**	30.1 (4.0)

**Figure 1:**
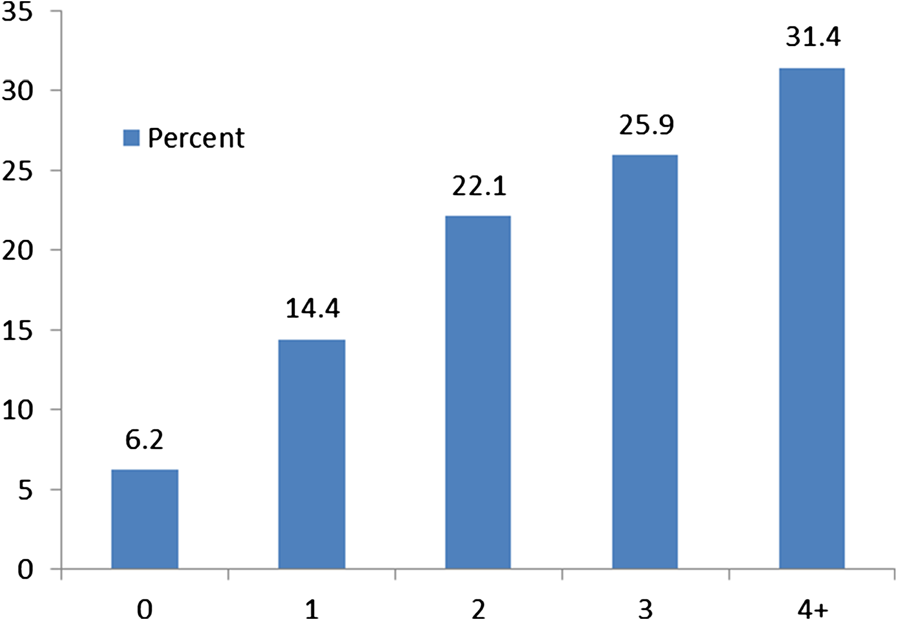
**Distribution of risk factors for PCPPP intervention group.**

As Figure 1 shows, 6 percent of women had no identified risk factors, 14 percent had only one risk factor, 22 percent had two risk factors, 31.4 percent had three risk factors and 25.9 percent had 4 or more major risk factors for a PTB. Prevalence rates for each of the major risk factors assessed are presented in Table 3. Of the women in the study sample 57.3 percent were diagnosed with urogenital tract infection, and 58.6 percent with periodontal disease. About 39 percent of women in the intervention group were identified as cigarette smokers. Just over 60 percent of the women had CES-D scores of >16; of these 81 or 17.2 percent were determined to be clinically depressed. A total of 106 or 22.5 percent of enrollees were determined to be reading at low levels of literacy. The assessment of housing conditions revealed that 392 of the women, or 83.2 percent, were living in an unstable housing situation.

**Table 3:** **Risk factor prevalence, acceptance rates and rates of minimal participation in PCPPP treatment protocols** ^1^

	**Prevalence/** **Eligible (a)**	**Acceptance ** **rate** ** (b)** ^**2**^	**Participation ** **rate** ^**3**^
	**N (%)**	**N (% of Eligibles)**	**N (% of Eligibles)**
***Intervention/*** ***Treatment***			
**Urogenital tract infection**	266 (57.3)	240 (90.2)	226 (85.0)
**Periodontal disease**	261 (58.5)	232 (88.8)	127 (48.7)
**Smoking**	185 (39.3)	106 (57.3)	52 (28.1)
**Clinical depression**	81 (17.2)	77 (95.1)	60 (74.1)
**Low literacy**	106 (22.5)	73 (68.9)	43(40.2)
**Housing instability**	392 (83.2)	357 (91.1)	259 (66.1)

^1^The number of women assessed (the denominator for prevalence) in all cases except for urogenital track infection and periodontal disease was 471. For periodontal disease 446 women were assessed by the dental hygienist; 25 women could not be assessed for periodontal disease because the hygienist experienced a temporary illness during the course of the study and was therefore unavailable at the time their clinic visit occurred. These 25 women were subsequently lost to follow-up. For urogenital tract infection 464 women were assessed/swabbed for infections; over the course of the study a total of 7 women had to leave the clinic before they could be tested for infections and were subsequently lost to follow-up**.**

^2^A women was classified as having accepted if she was identified as having a risk factor and agreed to receive the risk-appropriate treatment or service as defined in Table 1.

^3^See last column of Table 1 for explanation of how “participation” was defined.

Intervention/treatment acceptance rates are presented in column 2 of Table 3. In all cases, the majority of women was receptive and thus agreed to the respective treatment protocols. Specifically, of those diagnosed with a urogenital tract infection, 90.2% stated they were willing to accept treatment and took the medication in the presence of PCPPP staff. Acceptance rates for periodontal disease, clinical depression, literacy and housing were 88.6%, 95.1%, 68.9%, and 91.1%, respectively. In comparison, among those who were identified as smokers and therefore eligible for treatment, only 57.3% stated they were willing to accept treatment.

Participation rates, as percentage of women eligible for treatment varied considerably across the intervention arms of the study (Table 3: column 3). Exactly 85.0 percent of women diagnosed with urogenital tract infection met the definition of minimal participation described earlier, compared to 48.7 percent for periodontal disease. Among identified smokers, 28.1 percent participated by receiving some form of intervention designed to help women reduce or quit smoking. For the women with depressive symptomatology and who were subsequently determined to be clinically depressed the corresponding participation rate was 74.1 percent. The rate of participation for women identified with low literacy levels was 40.2 percent. Finally, among those women determined to be living in an unstable housing conditions, 66.1 percent participated by receiving some form of housing assistance.

The results of our analysis of differences in prevalence, acceptance and participation rates according to race/ethnicity and insurance status are presented in Table 4. Black (63.4%) and Hispanic women (49.4%) were far more likely than White women (28.9%) to have been diagnosed with a urogenital tract infection, and both Black and Hispanic women had much higher rates of periodontal disease than white women (61.5% and 69.0% vs. 23.8%, respectively). The assessments of health literacy revealed proportionally more black (24.0%) and Hispanic (24.7%) compared to white (8.7%) women with low literacy levels. Similarly, proportionately more black (85.8%) and Hispanic (84.4%) compared to white (58.7%) women were found to be living in unstable housing conditions. Women on Medicaid or without insurance were more likely than women with private insurance to have been diagnosed with depression (20.1 vs. 20.5%), as having low health literacy (26.2 vs. 14.1%), and as living in unstable housing conditions (88.1 vs. 71.8%). Where prevalence was significantly different according to both race/ethnicity and insurance status (periodontal disease, literacy, and housing conditions) the results of multiple logistic regression analysis indicated that the differences by race/ethnicity were independent of those for insurance status, and vice versa (data not shown, but available upon request).

**Table 4:** **Prevalence, acceptance rates and rates of participation: by race/ethnicity and insurance status** ^**1**^

		**Prevalence/Eligible**	**Accepted** ^**2**^ **treatment1**	**Participated** ^**3**^ ***(as % of Eligibles)***	
		**N (%)**	**N (%)**	**N (%)**	
**Infection**	White	13 (28.9)***	11 (84.6)	10 (76.9)	***Race/Ethnicity*** ^***1***^
	Black	210 (63.4)	189 (90.0)	178 (84.8)	
	Hispanic	38 (49.4)	36 (94.7)	34 (89.5)	
	Private	72 (51.8)	64 (88.9)	59 (81.9)	***Insurance Status***
	MA/Uninsured	194 (59.9)	176 (90.7)	167 (86.1)	
**Periodontal disease**	White	10 (23.8)***	4 (40.0)***	2 (20.0)	
	Black	198 (61.5)	183 (91.9)	102 (51.5)	
	Hispanic	49 (69.0)	41 (83.7)	21 (42.9)	
	Private	71 (51.8)	59 (83.1)	33 (46.5)	
	MA/Uninsured	189 (61.4)	173 (91.1)	94 (49.7)	
**Smoking status**	White	20 (43.5)	10 (50.0)	5 (25.0)	
	Black	132 (39.3)	75 (56.8)	37 (28.0)	
	Hispanic	32 (41.6)	20 (62.5)	9 (28.1)	
	Private	34 (23.9)***	18 (52.9)	12 (35.3)	
	MA/Uninsured	151 (46.2)	88 (58.3)	40 (26.5)	
**Clinical depression**	White	7 (15.2)	6 (85.7)	5 (71.4)	
	Black	53 (15.7)	50 (94.3)	40 (75.5)	
	Hispanic	19 (24.7)	19 (100)	13 (68.4)	
	Private	15 (10.5)**	14 (93.3)	12 (80.0)	
	MA/Uninsured	66 (20.1)	63 (95.5)	48 (72.7)	
**Housing instability**	White	27 (58.7)***	22 (81.5*)*	15 (55.6)	
	Black	289 (85.8)	265 (91.7*)*	192 (66.4)	
	Hispanic	65 (84.4)	61 (93.9)	44 (67.7)	
	Private	102 (71.8)***	85 (83.3)**	62 (60.8)	
	MA/Uninsured	289 (88.1)	271 (93.8)	197 (68.2)	
**Low literacy**	White	4 (8.7)	1 (25.0)	1 (25.0)	
	Black	81 (24.0)	57 (70.4)	31 (37.8)	
	Hispanic	19 (24.7)	13 (68.4)	10 (52.6)	
	Private	20 (14.1)**	9 (45.0)*	7 (35.0)	
	MA/Uninsured	86 (26.2)	64 (74.4)	36 (41.4)	

^1^There were 13 women who identified themselves as being something other than white, black or Hispanic; because of the small number they were not included in the figures for Race/Ethnicity shown here.

^2^A women was classified as having accepted if she was identified as having a risk factor and agreed to receive the risk-appropriate treatment or service as defined in Table 1.

^3^See last column of Table 1 for explanation of how “participation” was defined.

***p < .0001; **p < .01; *p < .05.

There were fewer instances where race/ethnicity and/or insurance status were significantly associated with rates of acceptance, with the general trend toward more willingness to accept treatment for minority and Medicaid or uninsured women. Specifically, Black (91.9%) and Hispanic (83.7%) women who were eligible for periodontal treatment were more likely to accept treatment than were White women (40.0%), while uninsured women or women on Medicaid were more likely than women with private insurance to accept the intervention for low literacy (74.4 vs. 45.0%), and more likely to accept help with housing problems (93.8 vs. 83.3%). Notably, as shown in column 3 of Table 4, there were no differences according to either race/ethnicity or health insurance status in terms of the rates of participation (among those who accepted treatment) for any of the treatment arms of the study, a finding which we discuss in more detail below.

## Discussion

The main purpose of the PCPPP trial was to determine if a set of systematic, coordinated, evidenced-based risk-reduction strategies implemented during the inter-conception period would reduce the risk of a subsequent PTB. We are unaware of any similar large scale RCT attempting to mitigate multiple known risk factors for repeat PTB, in a cohort of women in the immediate postpartum period following a premature delivery.

The findings presented here document the prevalence of the risk factors in the study cohort, the willingness of PCPPP enrollees to agree to receive treatment, and the extent to which women participated in the medical and other services offered as part of the PCPPP trial. As noted earlier, participation rates were calculated based on the lowest possible yet reasonable thresholds for classifying an enrollee as having ‘participated’. In addition, the PCPPP trial was designed to incorporate every reasonable effort to eliminate, or at least minimize all known barriers to accessing treatment or services.

Not surprisingly, given that the sample consisted of women who had just delivered prematurely, the prevalence of the risk factors in the study sample appear to be high when compared to available data for similar sociodemographic populations. Recent data available from the Centers for Disease Control (CDC) , for example, revealed that 24.7 percent of adults 30 to 34 years of age had mild to severe periodontal disease, and that rates increased markedly with age for both men and women [22]. The presence of periodontal disease in the CDC study was established according to the CDC/American Academy of Periodontology definitions, which are not directly comparable to ours. However, using a full-mouth examination and clinical confirmation by a periodontist we found that 58.5 percent of the PCPPP intervention group had moderate to severe periodontal disease. Pocket depth is generally considered an important marker for periodontal disease and all women identified as having periodontal disease in the PCPPP trial had at least one site with pocket depth ≥4 mm. By comparison, the detailed data from the above-mentioned CDC study showed that 29.6 percent of all adults 30–34 had at least one site with pocket depth >4 mm and that rates increased markedly with age. This is consistent with the conclusion that PCPPP women had a prevalence of periodontal disease considerably higher than that in the general population of women of similar age, especially in light of the fact that the average age of the PCPPP intervention group was only 25.6.

Data from the recent National Health Interview Survey reveal that about 21 percent of women 18–44 years age are current smokers [23], compared to almost 40 percent reported here. With respect to the prevalence of depression, reports tend to vary considerably depending on the screening instrument used, the cut-offs used to define depression and depression severity, or whether point or period prevalence rates are in question, among other things. Hence the precise ‘true’ prevalence of depression among postpartum women or women of childbearing age is unclear. A systematic review of the literature by Gavin and colleagues revealed that, approximately 19 percent of new mothers suffer from some form of depression at some point during the first three months following delivery, and as many as 7 percent have been diagnosed as having suffered from major depression [24]. In the PCPPP study a total of 471 women in the intervention group were screened for depressive symptomatology during the first 3 months postpartum; of those 237 or 29 percent were identified as being “possibly depressed” according to the Center for Epidemiological Studies of Depression Scale (CES-D). Subsequent evaluation of the “possibly depressed” group using the SCID revealed that 16 percent of the intervention group was clinically depressed, more than twice the equivalent period prevalence rate reported by Gavin and colleagues.

Urogenital tract infections screened for in the PCPPP study intervention included the more commonly reported conditions of bacterial vaginosis, chlamydia, and gonorrhea. The prevalence of these conditions in the study sample was 49, 10, and 1.7 percent, respectively, compared to 19, 2.2, and 0.24 percent reported elsewhere for young adult women or women of childbearing age in general [25-27].

In addition to the prevalence of the risk factors targeted for intervention in the PCPPP trial we also documented the rate at which eligible women were willing to participate in the respective interventions. With the exception of the smoking intervention the overwhelming majority of women identified with other risk factors expressed the willingness to receive the appropriate treatment or services. The analysis of participation patterns revealed that eligible women availed themselves of the various PCPPP treatments and services in widely varying degrees, ranging from a high of 85 percent (for infection) to a low of 28 percent (for smoking). Approximately 2 out of every 5 women identified with low literacy levels eventually received any related services, and fewer than half of the women with confirmed periodontal disease ever had any follow-up treatments. Again, in each case, every reasonable effort was made to remove or mitigate what are the most often-cited ‘barriers’ to receiving health care and related services -- including full reimbursement for costs and personalized accommodations related to transportation to and from clinical sites, free childcare, flexible and convenient clinical hours, and even home visits when necessary.

Not surprisingly, in most cases, the prevalence of risk factors was significantly higher for minority as compared to white women, and for low income women, as measured by insurance status. Black women enrolled in the study, for example, were more than two times more likely than white women to have been diagnosed with a urogenital tract infection and more than two and one-half times more likely to have been diagnosed with periodontal disease. In addition, both Black and Hispanic women were significantly more likely than white women to have reported housing instability, and were also significantly more likely to have elevated rates of low literacy.

There was no evidence to suggest that minority and low income women were either more reluctant to accept or able to take advantage of medical treatments or services offered as part of the PCPPP trial. *Where rates of acceptance differed to any degree they tended to be higher among minority as opposed to white women* (in the case of periodontal disease and low literacy) *or higher among women who were uninsured or on Medicaid, as compared to privately insured women* (in the case of housing instability*). Moreover, there were no significant differences in participation rates associated with either race/ethnicity or insurance status, pertaining to any of the treatments or services offered as a part of the PCPPP interventions.* The absence of any meaningful relationship between these socioeconomic factors and participation rates for the PCPPP trial suggests that the strategies used to make treatments and services as accessible as possible efforts were fairly successful.

The findings presented here also highlight the possibility of widely varying participation rates pertaining to interventions or programs offering multiple health and health-related treatments and services to women in the pre- or inter-conception period. Undoubtedly, participation will vary according to the perception of the benefits to those who enroll. Many other factors, however, will likely influence participation rates across ‘types’ of service or treatments. Ensuring that high-risk women will be able or willing to avail themselves of multiple appropriate treatments or services during the pre- or inter-conceptional period is increasingly being recognized as necessary to reduce the risk of PTB, since the underlying cause may be the cumulative effect of exposures and conditions which predate pregnancy, and may not be detectable or adequately addressed within the gestational period [2,6,7,28]. Unfortunately, the forces that motivate, enable, or inhibit women from accessing needed services outside the context of prenatal care, in the pre- or inter-conceptional period, are likely to be quite complex and are by no means fully understood. Successfully identifying the appropriate strategies for increasing access to and use of risk-reduction strategies and services may well require innovative and multi-level methodologies that match the complex nature of the problem.

## Conclusions

In summary, further research in this area appears warranted. We believe that we systematically and thoroughly addressed the well known and widely cited barriers associated with women’s access to and use of health-related services. Efforts to *maximize* participation of PCPPP enrollees were quite extensive, conducted as part of a fully-funded, large-scale randomized control trial to reduce repeat preterm birth. Nonetheless, participation rates varied and in some cases were quite low, even when defined in fairly minimal terms. This raises not only an important question about what more needs to be done in order to successfully deliver multiple high-quality risk-reduction interventions and services to high risk women, but also the relative costs and benefits of doing so. The more the chances of successfully reducing preterm birth rates hinge and are placed on interventions targeting high risk women prior to conception, the more important an adequate understanding of how to successfully reach such women with appropriate services and treatment becomes.

## Abbreviations

CDC: Center for disease control
CES-D: Center for Epidemiologic Studies Depression Scale
GA: Gestational age
GED: Graduate equivalency degree
PCPPP: The Philadelphia Collaborative Preterm Prevention Project
PTB: Preterm birth
SCID: Structured Clinical Interview for DSM-IV Dissociative Disorders
S-TOFHLA: Short test of functional health literacy in adults
TABE: Test of adult basic education
